# Multiparameter comparative analysis reveals differential impacts of various cytokines on CART cell phenotype and function *ex vivo* and *in vivo*

**DOI:** 10.18632/oncotarget.10510

**Published:** 2016-07-09

**Authors:** Xiao-Jun Xu, De-Gang Song, Mathilde Poussin, Qunrui Ye, Prannda Sharma, Alba Rodríguez-García, Yong-Min Tang, Daniel J. Powell

**Affiliations:** ^1^ Ovarian Cancer Research Center, Department of Obstetrics and Gynecology, Perelman School of Medicine, University of Pennsylvania, Philadelphia, PA, USA; ^2^ Department of Hematology Oncology, Children's Hospital of Zhejiang University School of Medicine, Hangzhou, Zhejiang, China; ^3^ Department of Pathology and Laboratory Medicine, Abramson Cancer Center, Perelman School of Medicine, University of Pennsylvania, Philadelphia, PA, USA

**Keywords:** chimeric antigen receptor, cytokine, adoptive immunotherapy, ovarian cancer, T cells

## Abstract

Exogenous cytokines are widely applied to enhance the anti-tumor ability of immune cells. However, systematic comparative studies of their effects on chimeric antigen receptor (CAR)-engineered T (CART) cells are lacking. In this study, CART cells targeting folate receptor-alpha were generated and expanded *ex vivo* in the presence of different cytokines (IL-2, IL-7, IL-15, IL-18, and IL-21), and their expansion, phenotype and cytotoxic capacity were evaluated, *in vitro* and *in vivo.* Moreover, the effect of the administration of these cytokines along with CART cells *in vivo* was also studied. IL-2, IL-7, and IL-15 favored the *ex vivo* expansion of CART cells compared to other cytokines or no cytokine treatment. IL-7 induced the highest proportion of memory stem cell-like CART cells in the final product, and IL-21 supported the expansion of CART cells with a younger phenotype, while IL-2 induced more differentiated CART cells. IL-2 and IL-15-exposed CART cells secreted more proinflammatory cytokines and presented stronger tumor-lysis ability *in vitro*. However, when tested *in vivo*, CART cells exposed to IL-2 *ex vivo* showed the least anti-tumor effect. In contrast, the administration of IL-15 and IL-21 in combination with CART cells *in vivo* increased their tumor killing capacity. According to our results, IL-7 and IL-15 show promise to promote *ex vivo* expansion of CART cells, while IL-15 and IL-21 seem better suited for *in vivo* administration after CART cell infusion. Collectively, these results may have a profound impact on the efficacy of CART cells in both hematologic and solid cancers.

## INTRODUCTION

Adoptive transfer of T cells genetically engineered to express chimeric antigen receptors (CARs) is an attractive strategy for cancer treatment. CAR-modified T (CART) cells redirected against tumor-associated antigens can mediate dramatic cancer remissions in acute lymphoblastic leukemia [1, 2], but less so in other cancers. Various strategies are being explored to further enhance the efficacy of CART cell therapy, including the incorporation of new signaling domains, co-expression of cytokine genes along with CARs, optimization of the conditions for CART cell expansion *ex vivo* and supplementation with exogenous cytokines [3, 4]. Additionally, the paradoxical finding that T cells with a less differentiated phenotypic and functional profile have an increased propensity to persist after infusion, generate memory and mediate cancer regression, has fostered efforts to generate, induce or selectively enrich T cells with these attributes [5–7]. The development of cell cultivation methodologies that yield such CART cells for clinic application is, accordingly, the source of active investigations.

Cytokines have important functions related to T cell expansion, differentiation, survival, and homeostasis. Common γ-chain (γ_c_) family cytokines are commonly used in clinical trials, and include interleukin (IL)-2, IL-4, IL-7, IL-9, IL-15 and IL-21 [8]. IL-2 has been the most widely studied as an immunotherapeutic agent for cancer and has shown to enhance the antitumor activity of CD19-specific CART cells in patients [9]. However, the administration of IL-2 has shown to be limited by adverse side effects, a propensity for the expansion of regulatory T cells and its impact on activated induced cell death (AICD) of T cells [10, 11]. IL-7, IL-15, and IL-21 can enhance the effectiveness of adoptive immunotherapies and appear to be less toxic as compared to IL-2 [12]. Among them, IL-7 and IL-15 have been reported to promote the induction and expansion of the human memory stem cell-like T cell subset, described as CD95+CD45RA+CD45RO+CD62L+CCR7+IL-7Rα+, that was able to engraft, expand and mediate enhanced activity in a xenogeneic transplant model of graft versus host disease (GVHD) [13]. In addition to γ-chain cytokines, IL-18 is an immunostimulatory IL-1 superfamily cytokine that regulates the immune response by enhancing the production of IFN-γ by T cells and natural killer cells, and augmenting the cytolytic activity of cytotoxic T lymphocytes [14]. Administration of IL-18 is safe and well-tolerated, even at a dose as high as 1000μg/kg [15], making it a good candidate to boost the antitumor activity of CART cells.

Although extensive preclinical and clinical studies have been performed on the role of many proinflammatory cytokines described above, a robust multi-parameter comparative study on the impact of the various exogenous γ_c_ cytokines on CART cell phenotype and function *in vitro* and *in vivo* is lacking. In this study, we have compared the effects of different γ_c_ cytokines and IL-18 on the expansion, phenotype and cytotoxicity of CART cells in order to identify the optimal cytokines for clinical use. The impact of the administration of those cytokines *in vivo* in combination with CART cells has been also explored in a xenograft mouse model.

## RESULTS

### Construction and expression of anti-FRα C4 CAR

**Figure 1:**
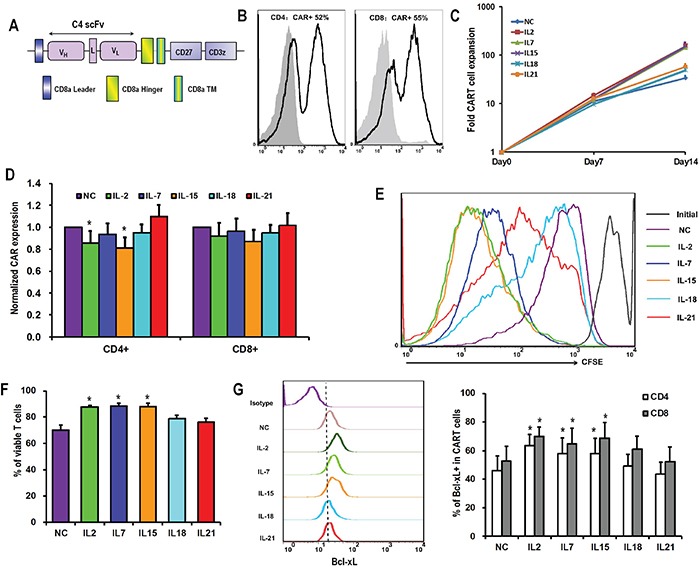
Differential effects of γc cytokines and IL-18 on CART cell accumulation **A.** Schematic diagram of C4-27z CAR vector. **B.** Representative flow histogram plot of CAR expression on CD4+ and CD8+ T cells 48 hours after lentiviral transduction. **C.** Overall accumulation of CART cells in response to various cytokines exposure. T cells were activated, transduced with lentivirus and exposed to various exogenous cytokines at final concentrations of 10ng/mL the next day (day 0). The numbers of CART cells were calculated based on the number of T cells and the percentages of CAR expression. The curves are representative from 6 donors. **D.** CAR expression by T cells 15 days after lentiviral transduction. The bar graph depicts CAR expression levels (± SEM, n=6) on the surface of CD4+ and CD8+ T cells, with the expression of CAR in no cytokine (NC) group normalized as 1. *P <0.05 versus NC group. **E.** Proliferation of T cells in response to various cytokines. On day 7 after lentivirus transduction, T cells in NC group were labeled with CFSE (2.5μM), and then exposed to various cytokines. Seven days later, T cells were analyzed for CFSE dilution by flow cytometry. **F.** Viability of T cells after 15 days of lentiviral transduction. T cells from various cytokine groups were stained with Annexin V and 7-AAD, and then analyzed for the proportions of viable cells (both Annexin V and 7-AAD negative). *P <0.05 versus NC group (n=6). **G.** Bcl-xL expression by CART cells. On day 15 after lentiviral transduction, CART cells were assessed for the expression of Bcl-2 protein by flow cytometry. The graph on the left is a representative flow histogram plot of Bcl-xL expression in various cytokine groups. The bar graph on the right depicts % of Bcl-xL positive CD4+ and CD8+ CART cells for 6 donors ±SEM. *P <0.05 versus NC group.

A lentivirus-based pELNS-C4-27z CAR plasmid was generated comprising the C4 human anti-folate receptor alpha (FRα) single-chain variable fragment (scFv) linked to the CD8α hinge and transmembrane regions, followed by a CD3ζ signaling moiety in tandem with the CD27 intracellular signaling motif (Figure 1A), and used for the generation of recombinant lentivirus. Primary human T cells were activated with anti-CD3/CD28 beads and transduced with the C4 CAR lentiviral vector with transduction efficiencies ranging from 43% to 65% when assessed 48h after transduction (Figure 1B). CAR expression levels were comparable between CD4+ and CD8+ T cells across multiple donors (52.6±10.2% vs. 49.5±17.1%, n=6, P=0.713).

### Influence of cytokines on the expansion of CART cells

The *in vitro* expansion of CART cells in the presence of various γ_c_cytokines and IL-18 was investigated. In the presence of IL-2, IL-7, or IL-15, activated and transduced CART cells expanded about 150 fold over the subsequent two weeks, while those grown with IL-18, IL-21 or in the absence of cytokines (no cytokine, NC) only expanded 30 to 50 fold (Figure 1C). CD4+ T cells exposed to IL-2 or IL-15 expressed lower CAR levels compared to the NC group (Figure 1D). Factors contributing to these expansion differences were investigated. Proliferative response was measured by monitoring cell division of CFSE-labeled T cells over the first seven days of culture with cytokines. As shown in Figure 1E, T cells cultured with IL-2 and IL-15 showed the greatest level of cell division, followed by IL-7. T cells from those groups also underwent significantly less apoptosis when compared with the no cytokine condition (Figure 1F). Consistent with reduced apoptosis, IL-2, IL-7, and IL-15 exposure resulted in the up-regulation of Bcl-xL expression both in CD4+ and CD8+ T cells, while this effect was not seen for IL-18 and IL-21 (Figure 1G).

### Influence of cytokines on the phenotype of CART cells

**Figure 2:**
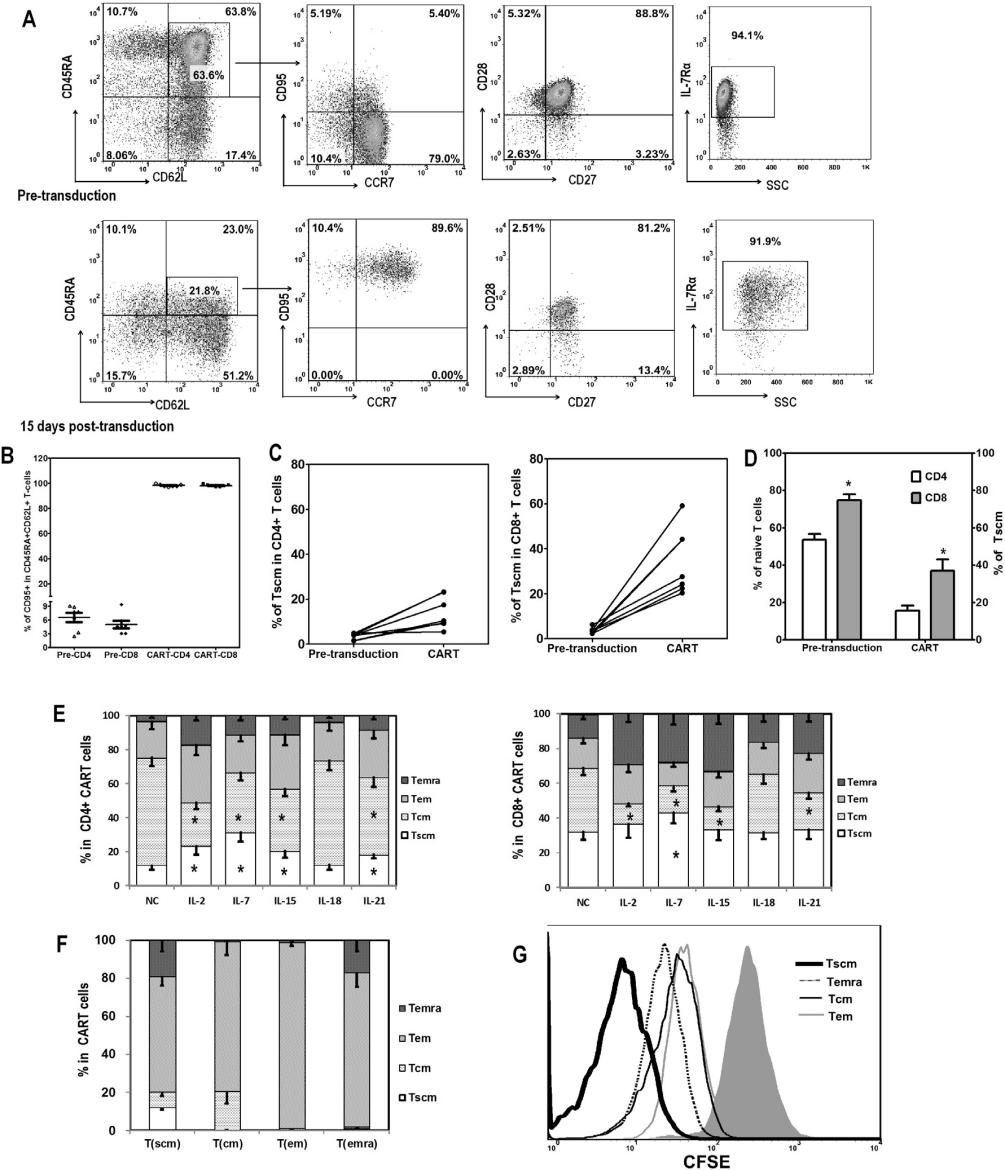
Memory T cell subsets of CART cells **A.** Representative flow plots showing the gating strategy of T cell subsets analysis are depicted. T cells were classified into four differentiation subsets based on CD45RA and CD62L expression: Tn (CD45RA+CD62L+), Tcm (CD45RA-CD62L+), Tem (CD45RA-CD62L-), and Temra (CD45RA+CD62L-). The expression of CD95, CCR7, CD27, CD28, and IL-7Rα was further evaluated for Tn to define Tscm subset (CD45RA+CD62L+CD95+CCR7+IL-7Rα+). **B.** CD95 expression in Tn subset (CD45RA+CD62L+) subpopulation of T cells before (Pre-CD4 and Pre-CD8) and 15 days after lentiviral transduction (CAR+T-CD4 and CAR+T-CD8). **C.** Increase of Tscm proportions in CD4+ and CD8+ T cells after lentiviral transduction. **D.** Correlation between the amount of Tn in pre-transduced T cells and the proportion of Tscm in CART cells after transduction, in CD4+ and CD8+ T cells. **E.** Distribution of differentiation subsets of CD4+ and CD8+ CART cells 15 days after lentiviral transduction and coculture with different cytokines. CD4+ and CD8+ T cells were first gated based on CAR and CD95 expression, then the CD95+ CART cells were divided into four subsets based on CD45RA and CD62L expression: Tscm (CD45RA+CD62L+), Tcm (CD45RA-CD62L+), Tem (CD45RA-CD62L-) and Temra (CD45RA+CD62L-). The high expression of CD27, CD28 and CCR7 was also confirmed in Tscm and Tcm subsets (data not shown). The proportions of Tscm were compared among various cytokine groups, *P <0.05 Tscm versus NC group (n=6). **F.** Self-renew and differentiation of different subsets of CART cells. FACS-sorted CAR+ Tscm, Tcm, Tem and Temra cells were cultured in the presence of IL-2 (10ng/mL) for 3 days, then analyzed for their phenotypes based on CD45RA and CD62L expression (n=3). **G.** Proliferation of various subsets of CART cells in response to IL-2. FACS-sorted CAR+ Tscm, Tcm, Tem and Temra cells were labeled with CFSE (2.5μM), and then cultured exposed to IL-2 (10ng/mL) for 3 days. Three days later, T cells were analyzed for CFSE dilution.

Fresh T cells from healthy donors were classified into four differentiation subsets based on CD45RA and CD62L expression: naïve T cells (CD45RA+CD62L+, Tn), central memory T cells (CD45RA-CD62L+, Tcm), effector memory T cells (CD45RA-CD62L-, Tem) and CD45RA positive effector memory T cells (CD45RA+CD62L-, Temra) (Figure 2A). The latter three were positive for CD95, while only a small portion of Tn expressed CD95 (3.6±1.4% in CD4+ and 3.7±1.3% in CD8+ T cells). Those cells also co-expressed CCR7, CD27, CD28, and IL-7Rα, and were referred to as memory stem T cells (CD45RA+CD62L+CD95+, Tscm). After activation and lentiviral transduction with CAR constructs, CD95 was greatly up-regulated to nearly 100% in Tn population (Figure 2B), thus increasing the percentage of T cells with Tscm phenotype, both in CD4+ and CD8+ CART cells, when compared to fresh T cells prior to transduction (Figure 2C). CD8+ CART cells had a higher percentage of Tscm cells than CD4+, which may be related to the higher proportion of the Tn subset in the starting CD8+ T cell population (Figure 2D).

The differentiation status of CART cells after fourteen days of co-culture with the different cytokines was then investigated based on the expression of CD45RA and CD62L in CAR+CD95+ T cells. As shown in Figure 2E, a significantly higher proportion of Tscm cells was found in the CD4+ CART cells after γ_c_ cytokine exposure compared to the no cytokine condition, with IL-7 treatment yielding the highest level of Tscm. In general, higher proportions of Tscm were found for CD8+ CART cells, compared to CD4+ in matched groups, and a higher percentage of the CD8+ Tscm subset was observed only in IL-7 treated CART cells (Figure 2E).

The ability of different CART cell subpopulations to self-renew and to differentiate into other cell subsets was assessed. CAR+ T cells were sorted based on CD45RA and CD62L expression into the four different differentiation subsets and then cultured separately in the presence of IL-2 for 3 days. Consistent with their differentiation status, Tscm were able to differentiate into all three other subsets, whereas Tcm and Temra were only able to differentiate into Tem (Figure 2F). When their proliferation abilities were further compared, we found that the Tscm subset exhibited a stronger proliferation capacity (Figure 2G). Furthermore, CD45RA expression inversely correlated with CFSE intensity, while CD62L and CCR7 expression showed no association with proliferation (Supplementary Figure S1).

**Figure 3:**
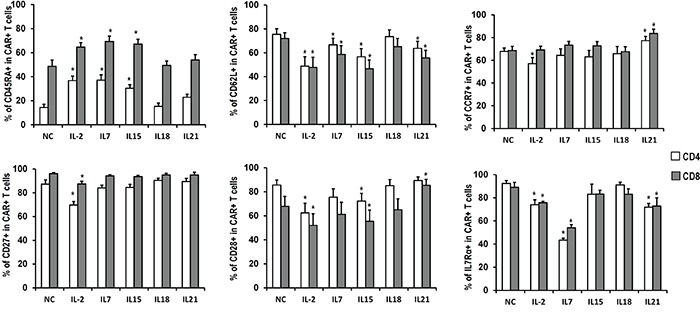
Phenotype of CART cells exposed to different cytokines Quantitation of CD45RA, CD62L, CCR7, CD27, CD28 and IL7Rα expression in CART cells expanded in the presence of indicated cytokines. Histograms represent mean values ± SEM from 6 independent donors. *P <0.05versus no cytokine (NC) group.

The expression of the surface markers CD45RA, CD62L, CCR7, CD27, CD28, and IL7Rα was also analyzed (Figure 3, Table 1). CD45RA was expressed in a higher portion of T cells exposed to IL-2, IL-7 or IL-15, as compared to the no cytokines group, which was consistent with the heightened proliferation and expansion of T cells cultured with these cytokines. CD62L expression in those groups, together with the IL-21 group, was significantly lower than in the control group. Furthermore, IL-2 down-regulated the expression of CCR7, CD27, CD28, and IL7Rα compared with cells expanded in the absence of exogenous cytokines. In addition, IL-7 down-regulated IL-7Rα expression, IL-15 down-regulated CD28 expression, and IL-21 down-regulated IL-7Rα while increasing CCR7 and CD28 expression. Altogether, these results suggest that IL-2 exposure induces the production of a more mature subset of T cells compared to IL-7, IL-15, or IL-21. In all cases, CART cells exposed to IL-18 showed a similar expression pattern to those without cytokine supplement.

**Table 1:** Effects of various cytokines on T-cell expansion, phenotype and function

	NC	IL2	IL7	IL15	IL18	IL21
**Expansion**						
T-cell expansion	0	++	+	++	0	0
CAR-T cell expansion	0	++	+	++	0	0
Bcl-xL expression	0	++	+	+	+	0
apoptosis	0	–	–	–	0	0
**Phenotype (without antigen challenge)**						
Tscm/CD4	0	+	++	+	0	+
Tscm/CD8	0	0	+	0	0	0
CD62L/CD4	0	– –	–	– –	0	–
CD62L/CD8	0	– –	–	– –	0	–
CD27/CD4	0	– –	–	–	0	0
CD28/CD4	0	– –	–	–	0	0
CD27/CD8	0	–	0	0	0	0
CD28/CD8	0	–	0	–	0	+
CD127/CD8	0	–	– –	0	0	–
CD127/CD8	0	–	– –	0	0	–
**Phenotype (with antigen challenge)**						
Tscm/CD4	0	0	0	0	0	0
Tscm/CD8	0	0	0	0	0	0
CD27/CD4	0	–	0	0	0	+
CD28/CD4	0	–	0	0	0	0
CD27/CD8	0	0	0	0	–	0
CD28/CD8	0	– –	– –	– –	–	+
**Function**						
Perforin	0	+	+	+	0	0
Granzyme B	0	+	0	+	–	++
IFNγ	0	0	0	0	0	0
TNFα	0	++	+	+	0	0
IL-2	0	0	0	0	0	0
CTL activity	0	+	+	+	0	+
**Anti-tumor effects in vivo**						
supplement before T-cell infusion	0	– –	0	0	0	0
supplement after T-cell infusion	0	+	+	++	0	++

### Influence of cytokines on the effector function of CART cells *in vitro*

**Figure 4:**
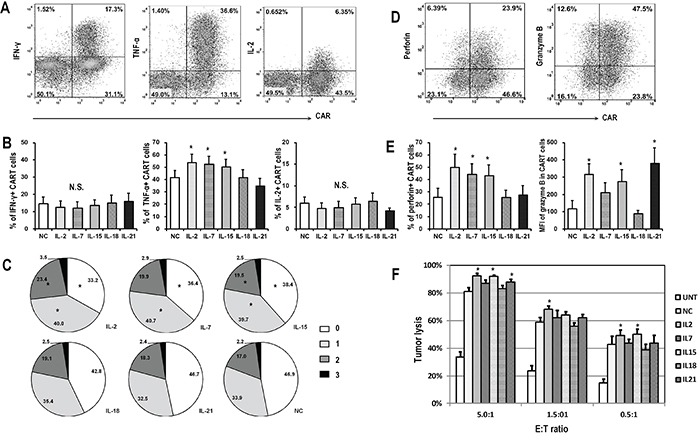
Functional analysis of CART cells exposed to different cytokines CART cells were exposed to indicated cytokines for 14 days, and then co-cultured with FRα+ SKOV3 cells for 5 hours before harvested for flow cytometry analysis. **A.** Representative flow plots showing the intracellular staining of IFN-γ, TNF-α and IL-2 in CART cells. **B.** Quantitative plots showing the percentages of cytokine-producing CART cells in various cytokine groups (n=6), *P <0.05 compared with no cytokine (NC) group. **C.** Pie charts depict the proportion of cells producing different numbers of cytokines (IFN-γ, TNF-α, and IL-2) after SKOV3 stimulation. *P <0.05 compared with NC group. **D.** Representative flow plots showing the expression of perforin and granzyme B in CART cells. **E.** Plots showing the quantification of perforin and granzyme B in CART cells exposed to various cytokines (n=6), *P <0.05 compared with NC group. **F.** Antigen specific cytotoxic activity of CART cells. Fourteen days after indicated cytokine exposure, the CART cells were assessed for cytolytic ability by using a luciferase-based assay after 18-hour of co-culture with SKOV3 target cells at the indicated E/T ratios. Untransduced T cells (UNT) served as negative effector controls. Data is shown as mean value ± SEM of 6 independent donors.

To investigate the influence of cytokines on CART cell effector function, the cytokine production capability of FRα-specific CART cells after stimulation with FRα+ SKOV3 cancer cells was first assessed. Following 5 hours of stimulation, IFN-γ, TNF-α, and IL-2 were detectable in the cytoplasm of 12.4-15.3%, 41.5-54.0%, and 4.3-6.5% of CART cells, respectively (Figure 4A and 4B). IL-2, IL-7, and IL-15 exposure induced more CART cells to produce TNF-α, while the frequencies of IFN-γ and IL-2 producing CART cells were comparable among all cytokine groups (Figure 4B). The fractions of responding CART cells were then tested for their polyfunctionality following the exposure to the different cytokines (Figure 4C). Compared to NC, the treatment with IL-2, IL-7, and IL-15 promoted more cytokine-producing CART cells, and conferred to those cells an increased potential to produce multiple cytokines following antigenic stimulation.

We next determined whether the expression of the cytolytic molecules, perforin and granzyme B, was affected by cytokine exposure (Figure 4D). Similar to TNF-α production, the exposure of CART cells to IL-2, IL-7, and IL-15 increased the expression of perforin and granzyme B, as compared to cells expanded in the absence of exogenous cytokines. Moreover, although CART cells exposed to IL-21 produced less TNF-α and perforin, they harbored the highest level of granzyme B, suggesting a possible increase in cytotoxic potential. Again, CART cells exposed to IL-18 presented a similar pattern than the NC group after antigenic stimulation (Figure 4E).

Finally, the specific tumor lysis activity of CART cells expanded in distinct cytokine conditions was quantified using a luciferase-based killing assay. As shown in Figure 4F, CART cells exposed to IL-2 or IL-15 lysed SKOV3 cells more efficiently than those exposed to IL-18 or NC, even at the lowest T cell to tumor ratio. To further confirm the association between the phenotype of CART cells and their function, T cells were sorted based on CAR and CD62L expression 14 days after lentiviral transduction and exposed to SKOV3 cells. CD62L+ CART cells (Tscm and Tcm) produced less cytokine and exhibited weaker cytolytic capacity when compared with CD62L- CART cells (Tem and Temra; Supplementary Figure S2).

### Expansion and phenotype of CART cells after antigen challenge

**Figure 5:**
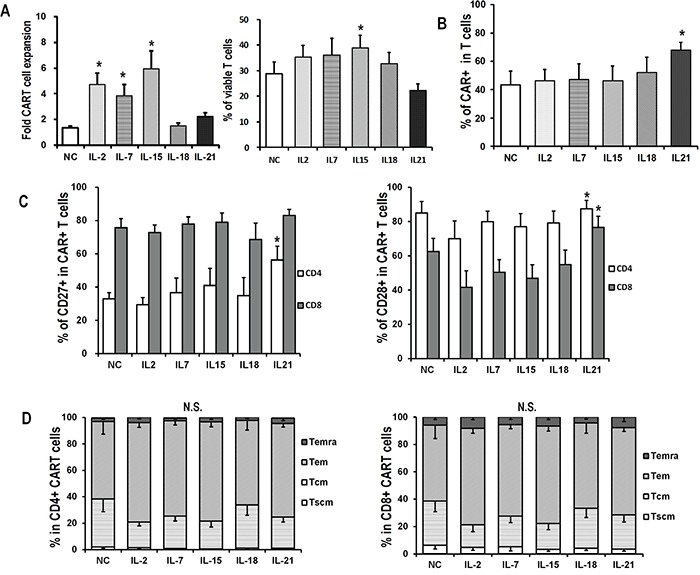
Expansion and phenotype of CART cells exposed to antigen challenge CAR-transduced T cells exposed to IL-2 were harvested on day 15, and then co-cultured with SKOV3 at an E/T ratio of 5:1 in the presence of indicated cytokines (10ng/mL) for 7 days **A.** Overall accumulation and viability of CART cells co-cultured with SKOV3 and indicated cytokines. Fold expansion is represented as mean value ± SEM. For viability analysis, T cells were stained with Annexin V and 7-AAD, and then analyzed for the proportions of viable cells by flow cytometry. *P <0.05 versus no cytokine (NC) group. **B.** CAR expression in IL-2-generated CART cells after 7 days of coculture with SKOV3 in the presence of indicated cytokines. *P<0.05 versus NC group. **C.** Quantitation of CD27 and CD28 expression on CART cells after 7 days of coculture with SKOV3 in the presence of indicated cytokines. *P<0.05 versus NC group. **D.** Distribution of memory T subsets of antigen-stimulated CD4+ and CD8+ CART cells in various cytokine groups. N.S., no statistical differences.

Exogenous cytokines have been injected after CART cell infusion in several clinical trials to enhance anti-tumor efficacy [16]. In order to identify candidate cytokines for its administration in combination with CART cells, we aimed to investigate the influence of various cytokines on the expansion of IL-2-generated CART cells following antigen encountering. CART cells were expanded in the presence of IL-2 for two weeks and then co-cultured with SKOV3 cells in the presence of alternative cytokines for 7 additional days and assayed for numerical expansion. Similar to the antigen-free conditions (Figures 1C and 1E), IL-2-generated CART cells which were exposed to antigen-expressing tumor cells and IL-2, IL-7, or IL-15 showed enhanced expansion over other cytokines, with IL-15-exposed cells showing the least apoptosis (Figure 5A). The frequency of CAR-expressing T cells was higher in the presence of IL-21 than other cytokines (Figure 5B), as well as CD27 and CD28 expression (Figure 5C). The differentiation status of antigen-stimulated CART cells was however different from cells in antigen-free conditions. Tscm cells were rarely found and Tem cells accounted for more than 50% of cells in all groups. In this setting of antigen stimulation, cytokines had no significant impact on the composition of memory T cell subsets, and IL-7 exposure did not favor the increase of Tscm cells (Figure 5D).

### Impact of prior cytokine exposure on CART cell anti-tumor efficacy *in vivo*

**Figure 6:**
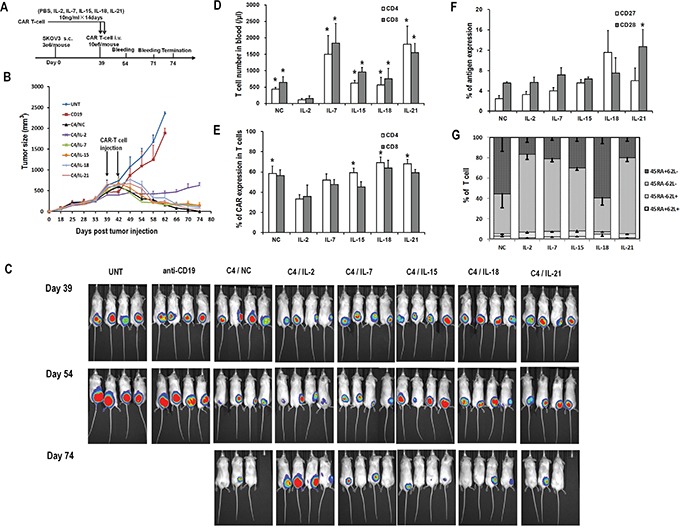
Antitumor activity of various CART cells with previous cytokine exposure **A.** *In vivo* experiment scheme. **B.** Tumor growth curves of mice treated with C4-27z CART cells, anti-CD19-27z CART cells and untransduced T cells. Data is presented as mean value ± SEM. Arrows indicate the time of T cell infusion. **C.** Bioluminescence images show fLuc+ SKOV3 tumors in NSG mice immediately before (day 38), two weeks (day 53) and five weeks (day 74) after first intravenous injection of CART cells. **D.** Quantitation of circulating human CD4+ and CD8+ T cell counts in mice peripheral blood 15 days after the first dose of CART cell infusion. *P<0.05 versus IL-2 group. **E.** Quantitation of CAR expression on circulating human CD4+ and CD8+ T cells in mice blood. *P<0.05 versus IL-2 group. **F.** Quantitation of CD27 and CD28 expression on circulating human T cells in mice blood. **G.** Distribution of T-cell subsets of circulating human T cells in mice blood based on CD45RA and CD62L staining.

To evaluate the effects of the various cytokines on the persistence and efficacy of CART cells *in vivo*, an immunodeficient mouse xenograft model of ovarian cancer was used. Mice bearing subcutaneous SKOV3 tumors were intravenously injected with two doses of C4-27z CART cells which had been previously exposed to the indicated cytokines for 2 weeks (Figure 6A). Tumor reduction occurred in all mice receiving CART cell infusions when compared with those animals injected with untransduced T cells or anti-CD19 CART cells (Figure 6B and 6C). Among the various *ex vivo* cytokine conditions, mice receiving CART cells with previous IL-2 exposure showed the least tumor control, consistent with the lowest number of circulating human T cells (Figure 6D). Tumors in mice receiving IL-7, IL-15, IL-18, IL-21, and NC exposed CART cells regressed or even disappeared, without any statistical difference in tumor size at the end point. In terms of persistence, IL-7 or IL-21 exposed CART cells accounted for higher total human T cell counts 15 days after the first CART cell infusion (Figure 6D). IL-15, IL-18, IL-21, and NC exposed CART cell groups all presented higher CD4+ CART cell frequencies when compared with the IL-2 exposed group, while the percentages of CD8+ CART cells were statistically comparable among all groups (Figure 6E). CD27 and CD28 markers were expressed only on about 5 to 10% of the circulating T cells and were comparable among all groups, except for that CD8+ T cells in the IL-21 group expressed higher CD28 than those in the IL-2 and NC groups (both P<0.05; Figure 6F). Most T cells were CD45RA-CD62L- but interestingly, IL-18 and NC exposure groups had much higher frequencies of circulating CD45RA+CD62L- T cells, in contrast to our findings in antigen-free conditions *in vitro* (Figure 6G and 2E). On day 32 after adoptive transfer, the circulating human T cells in all groups receiving CART cells expanded significantly except for in the IL-2 group, with average T cell counts of 242/μl in the IL-2 group and ranging from 14,907/μl to 19,651/μl in other groups (data not shown). By the end of the study, mice receiving untransduced or anti-CD19 CART cells required euthanasia due to tumor progression and two other mice (one in NC group and one in IL-21 group) were sacrificed due to graft-versus-host disease (GVHD)-like symptoms which can be associated with this model system, although their tumors had regressed.

**Figure 7:**
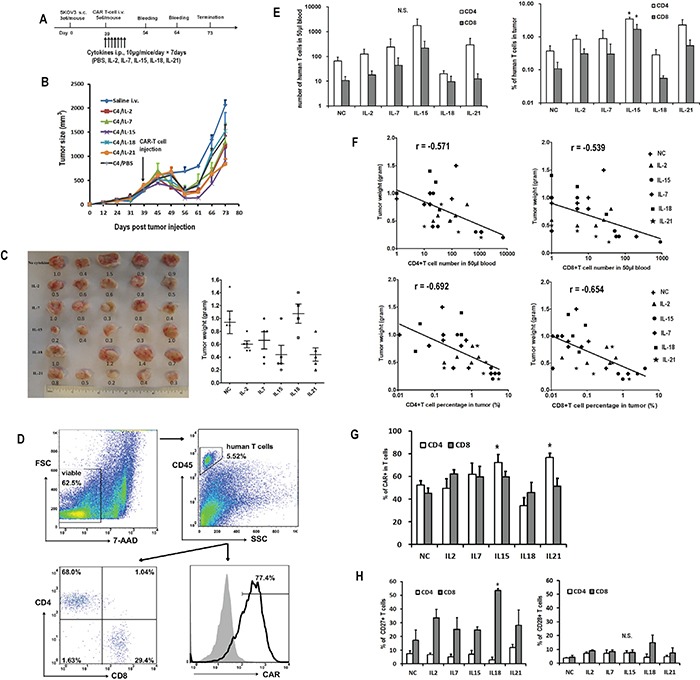
Impact of *in vivo* cytokine injection on the antitumor activity of CART cells **A.** *In vivo* experiment scheme. **B.** Tumor growth curves of mice treated with CART cells or saline. The data are presented as mean value ± SEM (five mice in each group, one mouse in IL-18 group died one day after T-cell injection). The arrow indicates the time of T cell infusion. **C.** Comparisons of tumor weights in various cytokine groups. The numbers in the left plot are the weight of the tumors. The lines in the right plots represent the mean±SEM. **D.** Representative flow plots showing the analysis of human T and CART cells in the tumor. The digested tumor tissues were stained with 7-AAD, CD45 PE, CD4 FITC and CD8 APC or biotin-SP-conjugated rabbit anti-human IgG (H+L) + streptavidin APC. **E.** Quantitation of circulating human CD4+ and CD8+ T cells in mice peripheral blood 15 days after T cell infusion and in tumor tissue at the end of the study. N.S., no significant difference. *P<0.05 versus no cytokine (NC) group. **F.** Negative correlation of tumor weight with the numbers of CD4+ (left) and CD8+ (right) T cells in peripheral blood and tumor tissue. **G.** Quantitation of CAR expression on human CD4+ and CD8+ T cells in tumors. **H.** Quantitation of CD27 and CD28 expression on human CD4+ and CD8+ T cells in mice tumor tissues. *P<0.05 versus NC group.

Next, we assessed whether the administration of cytokines after CART cell injection could enhance the anti-tumor activity of CART cells *in vivo*. Mice bearing established subcutaneous SKOV3 tumors received either single intravenous saline or suboptimal C4-27z CART cell injection on day 39, alone or in combination with daily intraperitoneal administrations of IL-2, IL-7, IL-15, IL-18 or IL-21 over one week (Figure 7A). Mice receiving CART cell therapy underwent transient tumor regression starting about 10 days after infusion, but the tumors began to rebound after day 56 (Figure 7B). Among the various cytokines, the combination of CART cells and IL-15 or IL-21 administration mediated the best anti-tumor response, followed by IL-2 and IL-7, whereas IL-18 and only CART cells treated mice had the heaviest tumor burden (Figure 7C). The persistence of transferred T cells in peripheral blood was determined 15 days after adoptive transfer and at study termination by flow cytometry. Highest numbers of CD4+ and CD8+ T cells were detected in mice treated with IL-15, though statistically significant differences were not detected (Figure 7E, left). At the end of the study, 34 days after T cell infusion, tumors were also analyzed for the presence of human CART cells (Figure 7D). Similar to what was observed in peripheral blood, mice treated with IL-15 had the highest number of T cells in the tumors (Figure 7E, right). CD4+ and CD8+ T cell counts, either in blood or tumor site, were both negatively associated with the final tumor weight (Figure 7F). Moreover, mice with more than 20 human CD4+ T cells/μL or 2 CD8+ T cells/μL of peripheral blood presented significantly higher tumor regression than others. Similarly, mice with more than 1% of human CD4+ and CD8+ T cells in tumors presented lower tumor burden. Frequencies of CAR expression in CD4+ and CD8+ T cells were comparable among most of the groups (45.1% to 62.4%), though IL-15 and IL-21 groups had significantly higher proportions of CAR-expressing CD4+ T cells compared to the NC group (Figure 7G). Finally, CD8+ T cells were generally more likely to retain CD27 expression, while CD28 expression was low and comparable between CD4+ and CD8+ T cells (Figure 7H).

## DISCUSSION

In the present study, a systematic comparative analysis of the effects of different γc cytokines and IL-18 on the *ex vivo* expansion, phenotype, and anti-tumor activity of CART cells *in vitro* and *in vivo* was conducted. Additionally, the effects of the direct *in vivo* administration of cytokines along with CART cells were investigated.

IL-2 is the most frequently used cytokine in generating lymphocytes for adoptive immunotherapy. We found that IL-2 significantly enhanced the accumulation of CART cells and their cytotoxic capacity *in vitro*. However, IL-2 exposed CART cells had inferior anti-tumor efficacy *in vivo* compared to T cells expanded in the presence of alternative cytokines. This finding demonstrates an inverse relationship between the effect of IL-2 on the cytolytic function *in vitro* and the *in vivo* tumor eradication, observation that is consistent with previous studies [17]. IL-2 exposed CART cells displayed a relatively mature phenotype, with low expression of CD62L, CCR7, CD27, and CD28, which correlated with a reduced blood persistence *in vivo* [18]. Recent studies have indicated that adoptive transfer of less differentiated T cells correlates with superior tumor regression, that aligns with our finding that IL-2 exposed CART cells are less effective *in vivo* [6, 19]. In addition, it is known that IL-2 favors the expansion of regulatory T cells that can inhibit the anti-tumor activity of the effector CART cells [10, 20]. However, further experiments are required to determine whether IL-2 manufactured CART cells have higher percentages of immunosuppressive regulatory T cells than other cytokine conditions.

IL-15 provided similar performance in stimulating CART cell expansion and tumor-lysis functions *in vitro* than IL-2, but induced a less differentiated phenotype, with higher CD27 and CD28 expression. Thus, preconditioning with IL-15 promoted a higher CART cell persistence *in vivo* along with improved antitumor immunity in tumor-bearing hosts.

Similar to IL-2 and IL-15, IL-7 showed a high ability to expand CART cells, but induced the highest proportion of Tscm subset in antigen-free conditions. However, these superiorities were not evident once CART cells were exposed to IL-7 after antigen challenge where a reduced expansion was observed. Consequently, IL-7 injection *in vivo* did not result in improved antitumor efficacy over IL-2 or IL-15.

IL-21 exerted few effects on CART cell accumulation as it could not enhance resistance to apoptosis by promoting Bcl-xL expression. However, it induced the expansion of less differentiated CART cells, with a CD62L+ CCR7+ CD27+ and CD28+ phenotype, even after antigen challenge. Therefore, IL-21 exposed CART cells showed the greatest persistence in animal models and the administration of this cytokine *in vivo* supported greater tumor eradication than other cytokines, excluding IL-15.

Lastly, IL-18 had little impact on CART cell expansion, phenotype and function both, *ex vivo* and *in vivo* compared to other cytokines or no cytokine supplementation.

An important finding of this study was that the effects of cytokine supplementation during *ex vivo* expansion or directly injected *in vivo* were quite different. In regards to the *ex vivo* expansion setting, we could not distinguish any superiority in terms of anti-tumor activity *in vivo* among IL-7, IL-15, IL-18, IL-21 and no cytokine (NC) conditions, perhaps due to CART cell number being sufficient to eliminate tumor in all the groups. However, IL-18, IL-21 or NC co-cultured cells had a limited expansion over time, which could be a limitation in terms of obtaining enough CART cells to be used *in vivo*. Therefore, our results implicate IL-15 and IL-7 as the best cytokines for clinical CART cell expansion. Of interest, a recent study showed that the combination of IL-7 and IL-15 supplementation instructs the generation of Tscm, which is beneficial to produce “younger” CART cells [13, 21]. As to *in vivo* cytokine administration, supplementation with all γ_c_ cytokines after antigen encountering appeared to favor the expansion of CART cells, with IL-15 being the one that provided the best support. Although IL-21 had little impact on CART cell accumulation, it seemed to favor the expansion of CART cells with “young” cytotoxic phenotype. This propensity may make it one of the better cytokines for *in vivo* supplement, along with IL-15. The moderate performance of IL-7 under antigen stimulation conditions *ex vivo* was consistent with its modest effect after injection to tumor-bearing hosts, although it induced higher frequencies of Tscm in antigen-free conditions.

Among the *in vivo* models, two mice in NC and IL-21 group with high amount of human T cell in peripheral blood died after tumor eradication and a few animals showed symptoms such as slightly arched back, hair ruffling, skin ulcer and diarrhea, which may be related to xenogeneic GVHD. In previous studies, persistent human chimerism has been associated with GVHD in conditioned NOD/SCID mice infused with human lymphocytes [13, 22], which is consistent with the present study. Thus, it remains possible that the anti-tumor activity of human CART cells may be in part supported by xenoreactivity in this model.

Regarding this particular study design, another aspect to take into account is that all the cytokines were evaluated at the same concentration. Even though the activity of all of them at the chosen concentration has been previously validated by others [17, 23, 24], it might not be the optimal for some and that can contribute to the observed differences. Therefore, further experiments testing whether these cytokines are working at plateau of their activity or not would be valuable to better interpret the results presented here.

Finally, which T cell properties are the most predictive of effective CART cell therapy is also an important issue deserving consideration. In terms of the differentiation status of CART cells, we and others have shown that CART cells with a “young” phenotype yield better antitumor activity than more differentiated T cells, that account for a shorter blood persistence [6, 21, 25]. Also the expansion of CART cells *in vivo* needs to be considered, since according to this study, mice with higher numbers of human T cells in blood and tumors had superior outcome. Then, CART cells with a less differentiated phenotype and with higher expansion capability would be the preferred for use *in vivo*.

In summary, our findings have important implications for the optimal production of CART cells and administration of exogenous cytokines to enhance the efficacy of CART cell adoptive therapy. Although widely used, IL-2 seems not to be the optimal strategy for CART cell expansion *ex vivo*. In contrast, IL-7 and IL-15 promote the expansion of CART cells with better properties before CART cell infusion, while IL-15 and IL-21 seem better suited for *in vivo* administration after CART cell infusion. However, as the cytokines have distinct impacts on T cell phenotype, expansion and function, experiments combining different cytokines are warranted to identify the best strategy to enhance the antitumor efficacy of CART cell therapy. On the other hand, the anti-tumor effect of T cells is influenced by many factors. For example, endogenous cytokines derived from the tumor microenvironment and regulatory cells including B regulatory cells and myeloid derived suppressor cells may impact the ultimate translation of these findings in the present study. Thus, whether the prioritization of cytokines suggested in this study will hold true in the presence of other immunoregulatory cell types *in vivo* should be further confirmed.

## MATERIALS AND METHODS

### CAR construction and lentivirus preparation

The pELNS-C4-27z CAR vector was constructed as described previously [26]. Briefly, the pHEN2 plasmid containing the anti-folate receptor alpha (FRα) C4/AFRA4 scFv was used as a template for PCR amplification of C4 fragment. The PCR product and the third generation self-inactivating lentiviral expression vectors pELNS were digested with BamHI and NheI. The digested PCR products were then inserted into the pELNS vector containing CD27-CD3z T-cell signaling domain. High-titer replication-defective lentivirus was generated by transfection of human embryonic kidney cell line 293T (293T) cells with four plasmids (pVSV-G, pRSV.REV, pMDLg/p. RRE and pELNS-C4-27z CAR) by using Express In (Open Biosystems) as described previously [26].

### T cells and cell lines

Peripheral blood lymphocytes were obtained from healthy donors after informed consent under a protocol approved by University Institutional Review Board at the University of Pennsylvania. The primary T cells were purchased from the Human Immunology Core after purified by negative selection. T cells were cultured in complete media (RPMI 1640 supplemented with 10% FBS, 100U/mL penicillin, 100μg/mL streptomycin sulfate) and stimulated with anti-CD3 and anti-CD28 mAbs-coated beads (Invitrogen) at a ratio of 1:1 following manufacturer's instructions. Twenty-four hours after activation, cells were transduced with lentivirus at MOI of 5. Indicated cytokines were added to the transduced T cells on the next day with a final concentration of 10ng/mL. The cytokines were replaced every 3 days. IL-2, IL-7, IL-5 and IL-21 were obtained from Peprotech, and IL-18 was obtained from MBL.

The 293T cell used for lentivirus packaging and the SupT1 cell used for lentiviral titration were obtained from ATCC. The established ovarian cancer cell lines SKOV3 (FRα+) was used as target cell for cytokine-secreting and cytotoxicity assay. For bioluminescence assays, SKOV3 was transduced with lentivirus to express firefly luciferase (fLuc).

### T cell expansion and cell division assay

To observe the accumulation of T cells, the cell density was counted by the Multisizer 3 Coulter Counter (Beckman Coulter). For proliferation assays, T cells were labeled with 2.5μM carboxyfluorescein succinimidyl ester (CFSE; Molecular Probes) for 5 minutes at 37°C and co-cultured with different cytokines at 12-well plate with an initial cell count of 1×10^6^/well for 7 days.

### Flow cytometric analysis and cell sorting

Flow cytometry was performed on a BD FACSCanto-II. Anti-human CD45 (HI30), CD3 (HIT3a), CD8 (HIT8a), CD45RA (HI100), CD62L (DREG-56), CCR7 (G043H7), IL-7Rα (A019D5), CD27 (M-T271), CD28 (CD28.2), CD95 (DX2), TNF-α (MAb11), IFN-γ (4S.B3), IL-2 (MQ1-17H12), perforin (B-D48), granzyme-B (GB11) were obtained from BioLegend. Biotin-SP-conjugated rabbit anti-human IgG (H+L) was purchased from Jackson Immunoresearch and APC conjugated streptavidin was purchased from Biolegand. Anti-human Bcl-xl (7B2.5) was purchased from Southern Biotech. Apoptosis kit and TruCount tubes were obtained from BD Bioscience. For peripheral blood T cell count, blood was obtained via retro-orbital bleeding and stained for the presence of human CD45, CD3, CD4 and CD8 T cells. Human CD45+-gated, CD3+, CD4+ and CD8+ subsets were quantified with the TruCount tubes following the manufacturer's instructions.

To isolate CAR-expressing Tscm, Tcm, Tem and Temra, CAR-transduced T cells were expanded in complete media for two weeks, then the T cells were stained with APC conjugated streptavidin, brilliant violet 421 anti-CD45RA antibody, PE anti-CD62L antibody and 7-AAD and sorted to >90% purity using FACSAria-II cell sorter (BD Biosciences). The sorted CART cells were then used for immunological assays.

### Intracellular protein analysis

T cells and SKOV3 cancer cells were plated at an E:T ratio of 1:1 (0.5×10^6^ effectors: 0.5×10^6^ targets) in 1ml of complete RPMI medium in a 24-well plate. Protein transport inhibitor GolgiPlug (BD Bioscience) was added to wells for intracellular cytokine staining experiments and the plate was incubated at 37°C for 5 hours (GolgiPlug was not added in perforin and granzyme-B analysis). The cells were collected and stained for cell surface markers (CD3 and CAR) and then were fixed and permeabilized using FoxP3 Fix/Perm Buffer (Biolegend), followed by staining for TNF-α, IFN-γ, IL-2, perforin or granzyme-B.

### ELISA assay for cytokine release

T cells and SKOV3 cells were plated at an E:T ratio of 1:1 (1×10^5^ effectors: 1×10^5^ targets) in 200μl of complete RPMI medium in a 96-well microplates. After 20-24 hoursof coculture, the supernatants were harvested and assayed for presence of IFN-γ using ELISA Kit (Biolegend) following manufacturer's instructions. Values represent the mean of triplicate wells.

### Cytotoxicity assays

For the cell-based bioluminescence assay, 2.5×10^4^ fLuc expressing SKOV3 cells were cultured with complete media in the presence of indicated ratios of transduced T cells with the use of in 96-well microplate. After incubation for 18 hours at 37°C, Luc-screen system reagents (Applied Biosystems) were added and incubated for 10min, then analyzed using a microtiter plate luminometer.

### *In vivo* studies

Eight to 12 week old female non-obese diabetic/severe combined immunodeficiency/γ-chain^−/−^(NSG) mice were obtained from the Stem Cell and Xenograft Core of the Abramson Cancer Center, University of Pennsylvania. The mice were inoculated subcutaneously with 3×10^6^ fLuc^+^ SKOV3 cells on the flank on day 0. After tumors became palpable mice were randomized in different treatment groups (n=4-5 mice/group). Human primary T cells for treatment were activated and transduced as described previously. In the first *in vivo* experiment (*ex vivo* cytokine), T cells were expanded without cytokine or in the presence of 10ng/mL of IL-2, IL-7, IL-15, IL-18 and IL-21, respectively for about 2 weeks. When the tumor size reached ~250-300 mm^3^, mice were injected with 5×10^6^ CART cells (~1×10^7^ T cells at 50% CART cell) intravenously twice with an interval of 3 days. The control groups of mice were injected with 5×10^6^ untransduced T cells or anti-CD19 CART cells twice. In the second *in vivo* experiment (*in vivo* cytokine), T cells were expanded in the presence of IL-2 (5ng/mL) for about 2 weeks. When the tumor size was ~250-300 mm^3^, the mice were injected with 5×10^6^ CART cells (~1×10^7^ T cells at 50% CART cell) or 100μl saline intravenously and then received daily intraperitoneal injection of 5μg of IL-2, IL-7, IL-15, IL-18, IL-21 or phosphate buffer solution (PBS) for 7 days. Tumor dimensions were measured with calipers and tumor volumes were calculated with the following formula: tumor volume = (length×width^2^)/2. The number and phenotype of transferred T cells in recipient mouse blood was determined by flow cytometry after retro-orbital bleeding. The mice were euthanized when the tumor volumes were ≥2000 mm^3^ and tumors were resected immediately for further analysis.

### Statistical analysis

Statistical analysis was performed with Prism 5 (GraphPad software) and IBM SPSS Statistics 20.0 software. The data were shown as mean ± SEM unless specified. Mann-Whitney U test was used for comparison of two groups. Findings were considered as statistically significant when P-values were less than 0.05.

## SUPPLEMENTARY FIGURES


