# Supplementary material for: Multiparameter comparative analysis reveals differential impacts of various cytokines on CART cell phenotype and function ex vivo and in vivo
Source: Oncotarget. 2016 Jul 9;7(50):82354–68. doi: 10.18632/oncotarget.10510 (PMC5347696; doi:10.18632/oncotarget.10510)
Supplement: Supplementary file 1 [file oncotarget-07-82354-s001.pdf]

Multiparameter comparative analysis reveals differential impacts of various cytokines on CART cell phenotype and function *ex vivo* and *in vivo*

SUPPLEMENTARY FIGURES

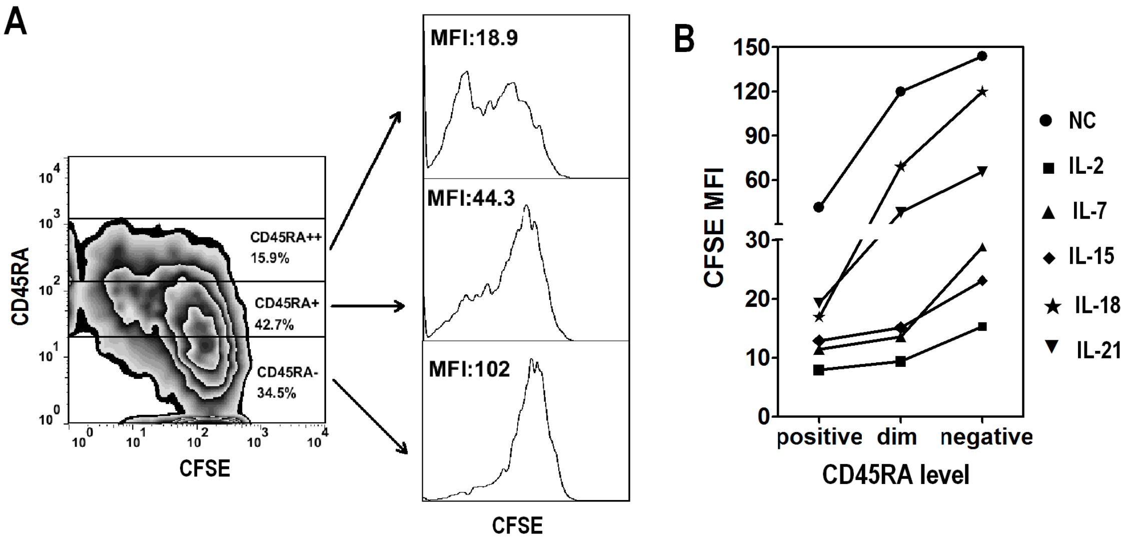

Supplementary Figure S1: CD45RA expression correlates with the proliferation of CART cells. A. Representative flow plots show highly proliferated CART cells presented high level of CD45RA expression. **B.** Quantification of CFSE mean fluorescence intensity (MFI) and CD45RA level in various cytokine groups.

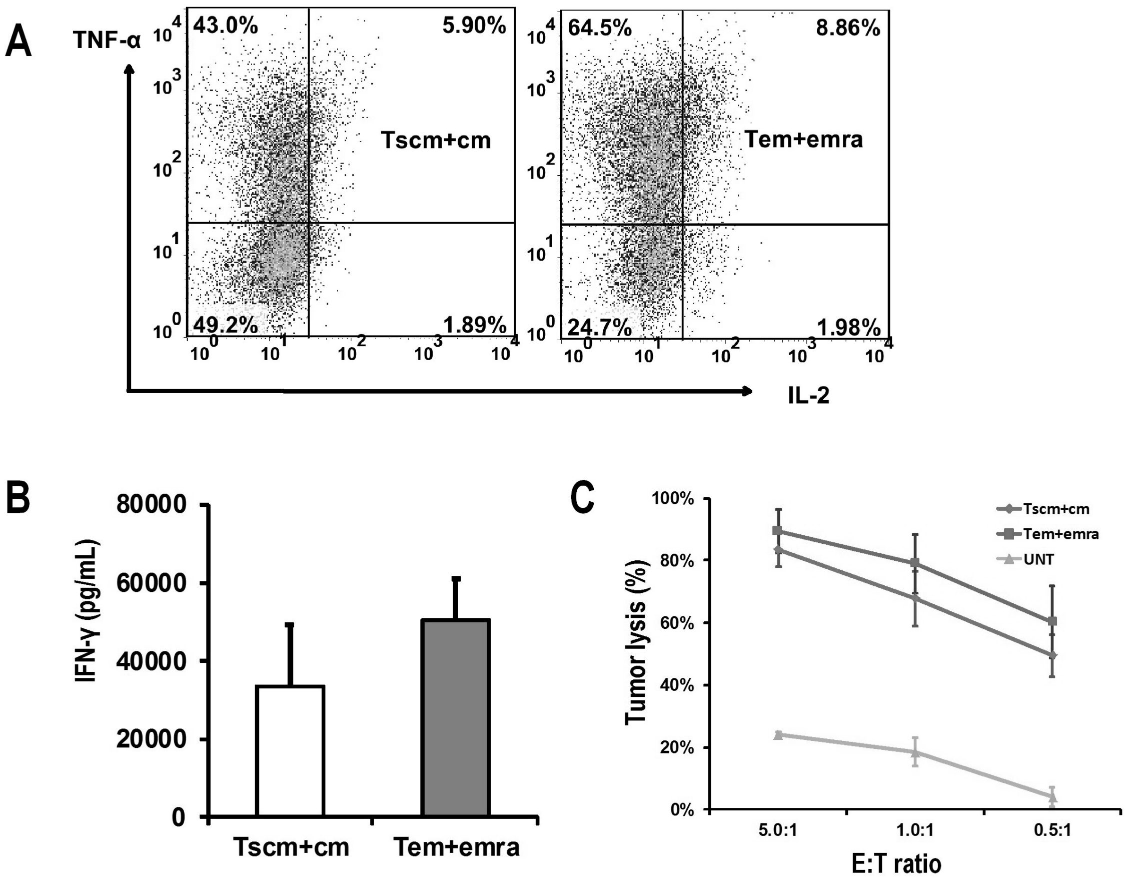

Supplementary Figure S2: Comparisons of cytokine-production and cytotoxicity between CD62L+ (Tscm and Tcm) and CD62L- (Tem and Temra) CART cells. The CD62L+ and CD62L- CART cell subsets were sorted from T cells exposed to IL-2 for 14 days and co-cultured with SKOV3 cells for 18-hours at indicated E/T ratios (n=3). **A.** The flow plot shows the TNF-α and IL-2 production, **B.** the histogram shows the IFN-γ secretion in the supernatant determined by ELISA, and **C.** the plot shows the tumor lysis activity.
